# Supplementary material for: Selective Functionalization of High-Resolution Cu2O Nanopatterns via Galvanic Replacement for Highly Enhanced Gas Sensing Performance
Source: Sensors (Basel). 2018 Dec 15;18(12):4438. doi: 10.3390/s18124438 (PMC6308504; doi:10.3390/s18124438)
Supplement: Supplementary file 1 [file sensors-18-04438-s001.pdf]

sensors

Supporting Information

Selective Functionalization of High-Resolution Cu_2O Nanopatterns via Galvanic Replacement for Highly Enhanced Gas Sensing Performance

Ju Ye Kim [1,2,†], Soo-Yeon Cho [1,2,†] and Hee-Tae Jung [1,2,*]

[1] Department of Chemical and Biomolecular Engineering (BK-21 Plus), Korea Advanced Institute of Science and Technology (KAIST), 291 Daehak-ro, Yuseong-gu, Daejeon 34141, Korea; juyekim@kaist.ac.kr (J.Y.K); chosooyeon@kaist.ac.kr (S.-Y.C)
[2] Korea Advanced Institute of Science and Technology (KAIST) Institute for NanoCentury, 291 Daehak-ro, Yuseong-gu, Daejeon 34141, Korea
† These authors contributed equally to this work.
* Correspondence: heetae@kaist.ac.kr; Tel.: +82-042-350-3931

Table of Contents

1. Schematic illustrations of expected galvanic reaction process.
2. Scanning electron microscope (SEM) observations depending on various reaction condition.
3. XRD spectrum of the Cu_2O nanopattern before and after thermal oxidation.
4. Photo and SEM image of the Cu_2O nanopattern sensors.
5. Elemental distribution analysis by transmission electron microscopy (TEM) - energy dispersive X-ray spectroscopy (EDS).
6. The composition analysis of over-galvanic reacted sample to confirm a selective decoration property of galvanic reaction.
7. Schematic of the overall gas delivery system.
8. NO_2 gas sensing performance comparison based on CuO or Cu_2O materials.
9. Response and recovery time of the Cu_2O/Pt nanopattern sensor to 1-4 ppm NO_2.
10. Selectivity characterization of the Cu_2O/Pt nanopattern sensor.
11. Long-term stability of the Cu_2O/Pt nanopattern sensor.

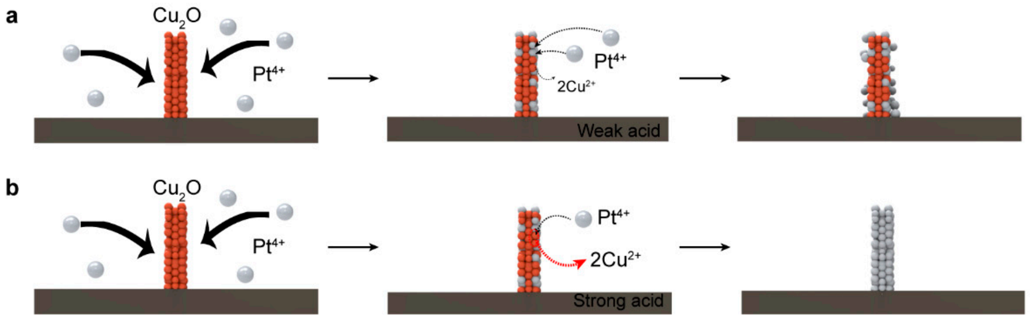

Figure S1. Schematic illustrations of expected galvanic replacement process. **(a)** When the reaction occurs in weak acid condition, both Pt reduction on Cu_2O surface and replacement for Cu_2O occur due to the mild limitation of galvanic reaction. On the other hand, **(b)** in strong acid condition without adding NaOH, galvanic reaction process predominates resulting in complete replacing Cu_2O with Pt.

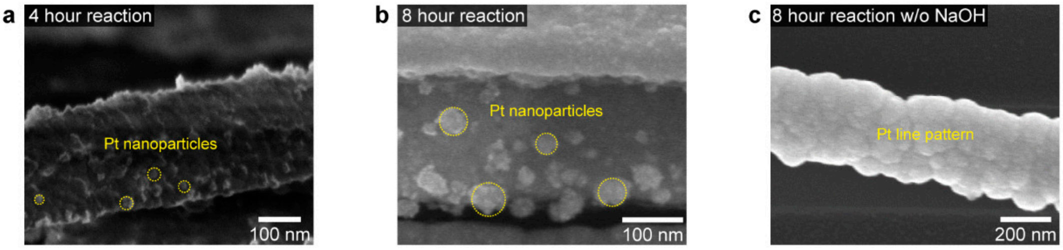

Figure S2. SEM images of **(a)** 4 hour reaction in pH 5 solution (with NaOH), **(b)** 8 hour reaction in pH 5 solution (with NaOH) and **(c)** 8 hour reaction in pH 2 solution (without NaOH). In a mild galvanic reaction condition, Pt particle aggregation occurs allowing the reduction of Pt rather than replacing Cu_2O with Pt. In a strong condition, however, all Cu_2O replaced into Pt wire.

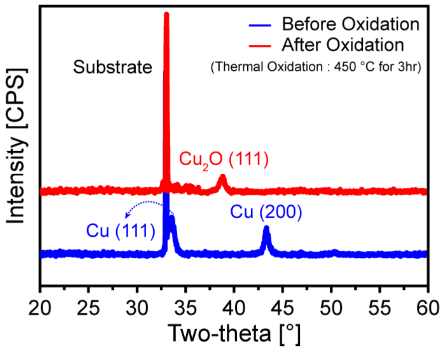

Figure S3. XRD spectrum of the Cu_2O nanopattern before and after thermal oxidation.

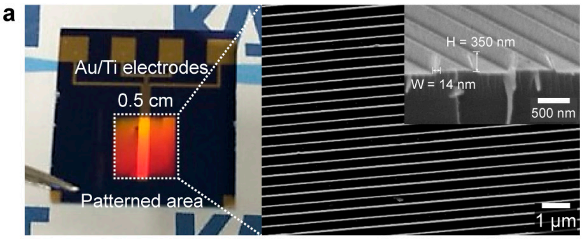

Figure S4. Photo and SEM image of the Cu_2O nanopattern sensors.

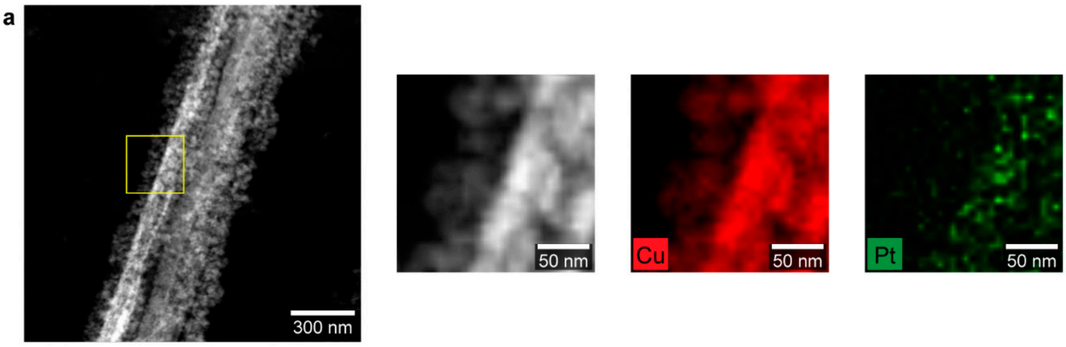

Figure S5. Elemental distribution analysis of the fabricated Pt/Cu_2O pattern array by energy-dispersive X-ray spectroscopy (EDS). **(a)** Left figure shows high-angle annular dark-field scanning transmission electron microscopy (HAADF-STEM) image of selectively Pt functionalized Cu_2O nanopattern and its elemental mapping analysis is shown sequentially.

TEM Sample Preparation

For preparation of TEM sample, poly(methylmethacrylate) (PMMA, Sigma-Aldrich) was coated on synthesized Cu_2O line pattern on SiO_2 substrates (3000 rpm, 45 s). After thermal treatment at 180°C for 3 min to evaporate the solution, the substrate was immersed on potassium hydroxide (KOH) 2M solution at 95°C for separating the line pattern from the substrate. After 2~3 hours, line pattern film was floating which means separation from the SiO_2 substrate. Once the film was cleaned with acetone and water, it was transferred to Ni TEM grid and washed with acetone once again and then dried in the oven overnight.

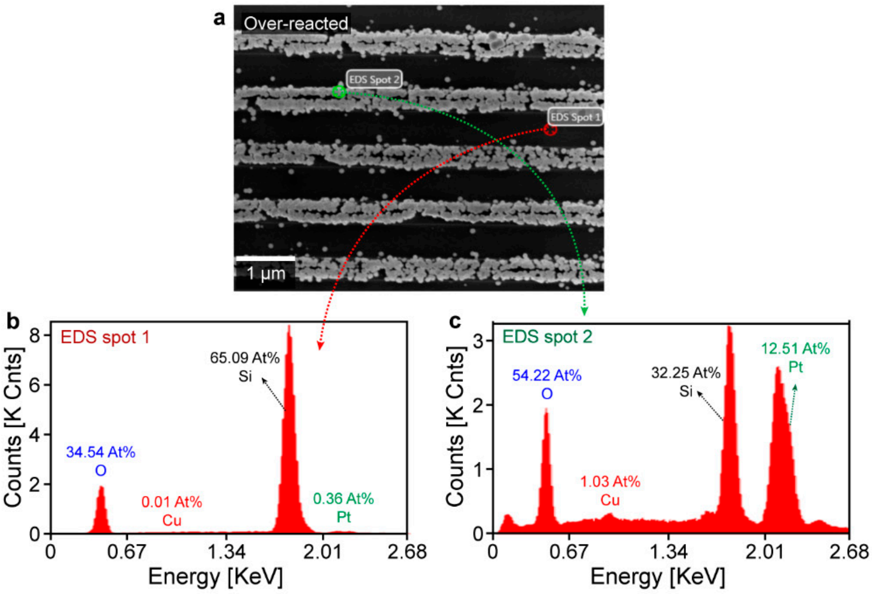

Figure S6. The composition analysis of over-galvanic reacted sample (complete reaction from Cu_2O to Pt) in low magnification. **(a)** Scanning electron microscopy (SEM) image in low magnification. Point spot energy-dispersive X-ray spectroscopy (EDS) analysis for **(b)** the space between the line patterns and **(c)** line pattern surface itself.

Table S1. NO_2 gas sensing performance comparison based on CuO or Cu_2O materials.

Sensing Material	NO_2 concentration (ppm)	Sensor response	Response definition	Operating temperature (°C)	ref
Cu_2O thin film	1.5	3	$(R_g-R_o)/R_o$	150	1
CuO particulates	50	0.036	$(R_a-R_g)/R_g$	150	2
CuO nanowires	4	1.17	R_g/R_a	~370	3
CuO plates	50	2.2	R_a/R_g	200	4
p-CuO nanowires	10	0.55	$(R_g-R_o)/R_o$	300	5
2.2 Cr-CuO	100	134.2	R_a/R_g	250	6
Cu_2O/CuO-10	0.001	13.5	$(I_g-I_a)/I_a$	RT	7
Pt/Cu_2O ultrahigh nanopattern	4	2.75	R_a/R_g	300	This work

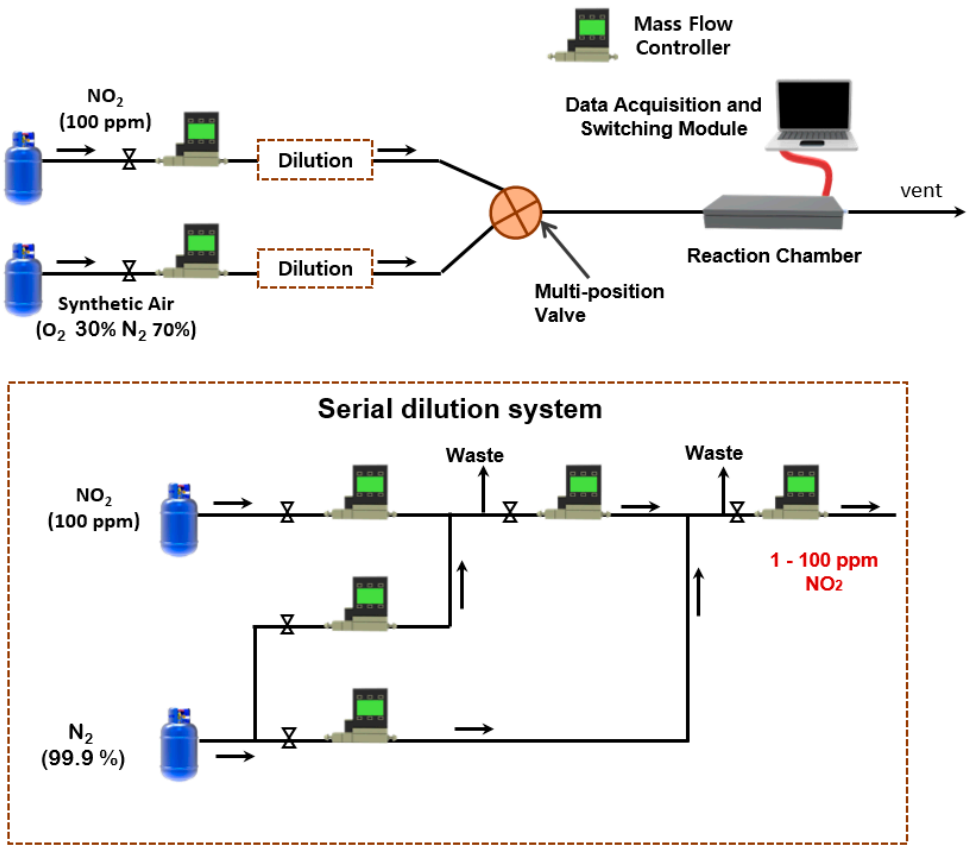

Figure S7. Schematic of the overall gas delivery system. NO2 and synthetics air was introduced in a controlled manner into the reaction chamber by using the MFC, tubing system, and multi-position valve. The serial dilution system was also used to obtain 1–100 ppm concentrations of the NO2.

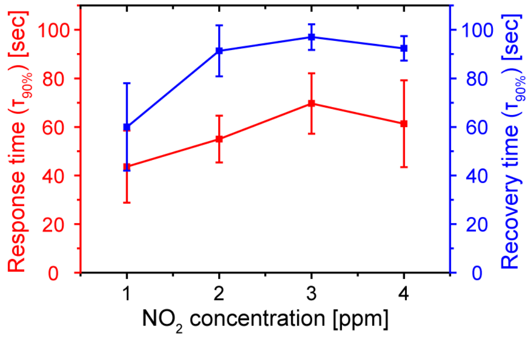

Figure S8. Response and recovery time of the Cu2O/Pt nanopattern sensor to 1-4 ppm NO2.

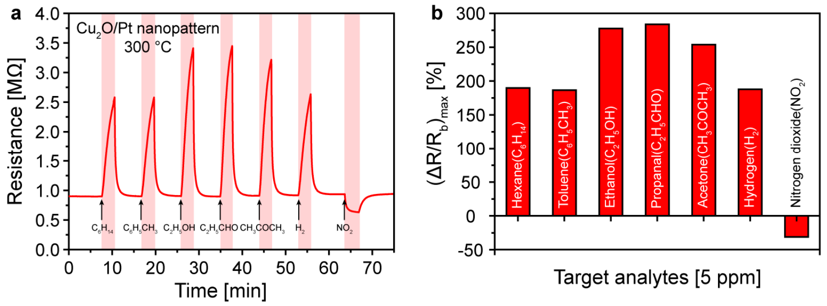

Figure S9. Selectivity characterization of the Cu2O/Pt nanopattern sensor. (a) Real-time response behavior of the sensors to various analytes including hexane, toluene, ethanol, propanal, acetone, hydrogen, NO2 (all 5 ppm). (b) Maximum response amplitudes of the sensors to each analytes.

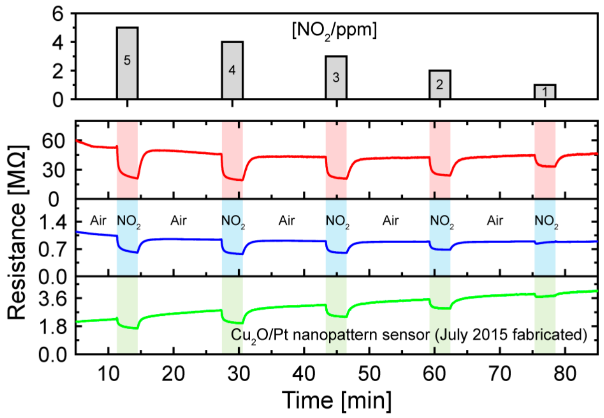

Figure S10. Long-term stability of the Cu_2O/Pt nanopattern sensor (Real-time sensing signals to 1-5 ppm NO_2 with sensors fabricated three years ago).

References

1. Shishiyanu, S.T.; Shishiyanu, T.S.; Lupan, O.I. Novel NO_2 gas sensor based on cuprous oxide thin films, *Sens. Actuators, B.*, **2006**, 113, 468–476, doi: 10.1016/j.snb.2005.03.061.
2. Das, A.; Venkataramana, B.; Partheephan, D.; Prasad, A.K.; Dhara, S.; Tyagi, A.K. Facile synthesis of nanostructured CuO for low temperature NO_2 sensing, *Phys. E.*, **2013**, 54, 40–44, doi: 10.1016/j.physe.2013.06.007.
3. Kim, Y.-S.; Hwang, I.-S.; Kim, S.-J.; Lee, C.-Y.; Lee, J.-H. CuO nanowire gas sensors for air quality control in automotive cabin. *Sens. Actuators, B.*, **2008**, 135, 298–303, doi: 10.1016/j.snb.2008.08.026.
4. Li, Y.; Liang, J.; Tao, Z.; Chen, J. CuO particles and plates: Synthesis and gas-sensor application, *Mater. Res. Bull.*, **2008**, 43, 2380–2385, doi: 10.1016/j.materresbull.2007.07.045.
5. Kim, J.-H.; Katoch, A.; Choi, S.-W.; Kim, S.S. Growth and sensing properties of networked p-CuO nanowires, *Sens. Actuators, B.*, **2015**, 212, 190–195, doi: 10.1016/j.snb.2014.12.081
6. Kim, K.-M.; Jeong, H.-M.; Kim, H.-R.; Choi, K.-I.; Kim, H.-J.; Lee, J.-H. Selective Detection of NO_2 Using Cr-Doped CuO Nanorods, *Sensors.*, **2012**, 12, 8013–8025, doi: 10.3390/s120608013
7. Hu, J.; Zou, C.; Su, Y.; Li, M.; Han, Y.; Kong, E.S-W.; Yang, Z.; Zhang, Y. An ultrasensitive NO_2 gas sensor based on a hierarchical Cu_2O/CuO mesocrystal nanoflower. *J. Mater. Chem. A.*, **2018**, 6, 17120-17131, doi: 10.1039/c8ta04404j.

© 2018 by the authors. Licensee MDPI, Basel, Switzerland. This article is an open access article distributed under the terms and conditions of the Creative Commons Attribution (CC BY) license (http://creativecommons.org/licenses/by/4.0/).
